# The Relationship Between Emotional Intelligence and Job Performance Among Critical Care Nurses: A Cross-Sectional Study

**DOI:** 10.3390/healthcare14040442

**Published:** 2026-02-10

**Authors:** Saud Abdullah Aljanfawi, Richard Balacuit Maestrado, Bader Emad Aljarboa, Nashi Masnad Alreshedi, Bander Abdullah Aljanfawi, Ibrahim Alasqah, Abdullelah Modhi Alsolais, Joyce Batuyog Buta, Omar Hamed Alshammari, Fahad Bader Fahad Alhazmi, Khadijah Abiodun Okusanya, Afnan Hamad Alshammari

**Affiliations:** 1 Nursing Administration Department, King Salman Specialist Hospital, Masyaf District, Hail 55471, Saudi Arabia; saaljanfawi@moh.gov.sa (S.A.A.); baljarboa@moh.gov.sa (B.E.A.); baljanfawi@moh.gov.sa (B.A.A.); 2 Medical-Surgical Nursing Department, College of Nursing, University of Hail, Hail 81451, Saudi Arabia; 3 Hail Health Cluster, Ministry of Health, Hail 55471, Saudi Arabia; nmalreshidi@moh.gov.sa; 4 Department of Community, Psychiatric and Mental Health Nursing, College of Nursing, Qassim University, Buraydah 52571, Saudi Arabia; i.alasqah@qu.edu.sa; 5 College of Applied Medical Sciences, Shaqra University, Riyadh 11421, Saudi Arabia; aalsolais@su.edu.sa; 6 Department of Maternal and Child Health Nursing, College of Nursing, Qassim University, Buraydah 52571, Saudi Arabia; j.buta@qu.edu.sa; 7 Intensive Care Unit, King Salman Specialist Hospital, Masyaf District, Hail 55471, Saudi Arabia; omhalshammari@moh.gov.sa (O.H.A.); fabalhazmi@moh.gov.sa (F.B.F.A.); kokusanya@moh.gov.sa (K.A.O.); aalshammari298@moh.gov.sa (A.H.A.)

**Keywords:** emotional intelligence, job performance, critical care nurses, emotional intelligence scale, health care, Saudi Arabia

## Abstract

**Introduction:** Emotional intelligence (EI) is increasingly acknowledged as a component that may influence nurses’ job performance (JP), particularly in high-stress contexts. This study examined the relationship between emotional intelligence and job performance among critical care nurses at King Salman Specialist Hospital in Hail, Saudi Arabia. **Design/Methods:** The cross-sectional study included 50 registered nurses working in the critical care unit, following the Strengthening the Reporting of Observational Studies in Epidemiology (STROBE) checklist. Data were gathered using validated tools. The data were collected between October and December 2024. Point–biserial correlation (rpb), one-way ANOVA and simple linear regression were employed. **Results:** This study found that neither gender (r_pb_ = 0.095, *p* = 0.514) nor age group (F = 0.945; *p* = 0.423) had a significant impact on EI or JP scores. Meanwhile, the linear regression model was highly significant (F [1, 48] = 45.829; *p* < 0.001), indicating that EI is a robust predictor of performance in this cohort. Contrary to common assumptions, a significant negative (inverse) relationship was identified. For every one-unit increase in EI, job performance decreased by 0.541 units (β = −0.699; t = −6.77; *p* < 0.001). **Conclusions:** This study confirms that EI serves as a notable inverse predictor of JP of critical care nurses. This shows that there could be high levels of emotional labor in the demanding clinical environment, which could hinder technical performance. This finding, irrespective of age or gender, defies the ‘more is better’ generalization of EI in the healthcare industry. Therefore, it is essential that there be available supportive mechanisms in the workplace to assist nurses with high EI in managing their emotional involvement with clinical work. This should be done to avoid a compromise in job performance.

## 1. Introduction

Nursing is widely recognized as a demanding and emotionally taxing profession. Nurses are exposed to numerous daily stressors, including excessive workload, interpersonal conflicts, rotating shifts, patient deaths, and persistent stress [1]. Despite these challenges, nurses are responsible for delivering comprehensive care aimed at improving and maintaining patients’ health, which encompasses complete physical, mental, and social well-being [2,3]. Within this holistic framework, emotional intelligence (EI) has gained prominence as a critical psychological resource. Cognitive intelligence contributes approximately 20% to individual performance. EI is defined as the ability to perceive, regulate, and utilize emotions to promote growth and is a significant influencing factor regarding efficacy in high-stress professions [1,4].

Recent research underscores that nurses require not only clinical skills but also the capacity to manage emotions effectively to enhance patient satisfaction and clinical outcomes [5,6]. Evidence suggests that EI influences decision-making under pressure; individuals with high EI demonstrate resilience, while those with lower EI are more susceptible to stress that impairs clinical adaptation [7]. Global studies have established a strong correlation between EI and nursing performance. For instance, research among ICU nurses in Poland found that those with higher EI utilized active coping and planning more frequently while exhibiting lower rates of behavioral disengagement [8]. Similarly, a study in Shiraz, Iran, identified a significant inverse relationship between EI and job stress, suggesting that EI training could mitigate workplace tension [1]. In the healthcare industry, high EI is critical for communication and problem-solving [4]. While emotional labor can sometimes negatively impact job satisfaction, it has been shown to have a direct beneficial effect on job performance when managed correctly [9]. Furthermore, EI has been linked to increased job well-being and higher occupational engagement as nurses with higher EI provide more compassionate care and report better teamwork [2,5,10]. In the Middle East, the nursing workforce is unique, with 60–70% of hospital nurses being expatriates from diverse cultural backgrounds [11]. This multinational composition necessitates an exploration of how EI is shaped by sociocultural factors and applied in a region with specific stressors. Despite the established broader links between EI and performance, there remains a significant gap in focused investigations concerning critical care nurses in Saudi Arabia. Understanding their emotional experiences is essential to optimizing care quality in high-intensity environments like intensive care units (ICUs) [4,12].

This study is guided by a framework where EI facets—self-awareness, self-regulation, self-motivation, social awareness, and social skills—are hypothesized to enhance job performance [13]. Self-motivation drives goal attainment, while empathy fosters the mutual trust necessary for organizational commitment [10]. Based on this framework and the existing literature, this study proposes two primary hypotheses, which include: emotional intelligence improves job performance among critical care nurses; and there is a positive link between emotional intelligence and occupational engagement. This study aims to determine the relationship between EI and job performance among critical care nurses at King Salman Specialist Hospital in Hail, Kingdom of Saudi Arabia. To achieve this, the study addresses the following objectives: to assess the specific relationship between EI and job performance within this high-stress critical care cohort; and to determine the socio-demographic characteristics (such as age, gender, and experience) that are significantly associated with EI and job performance in this setting.

## 2. Materials and Methods

### 2.1. Study Design

A cross-sectional observational study design was employed to examine the relationship between emotional intelligence (EI) and job performance. This design provides a “snapshot” of outcomes and exposures within the study population at a single point in time [14]. The Strengthening the Reporting of Observational Studies in Epidemiology (STROBE) checklist was used to guide reporting in the study (see Appendix A).

### 2.2. Setting

The study was conducted at King Salman Specialist Hospital (KSSH) in Hail, Saudi Arabia. The KSSH is a tertiary-level specialist hospital that serves as a major referral center for the Hail Region, providing care to a reference population of approximately 700,000 residents. The intensive care units (ICUs) at KSSH are high-intensity clinical environments characterized by complex care requirements and high emotional demands, making them a relevant setting for investigating EI in nursing.

### 2.3. Sample and Sampling

The study utilized purposive total population sampling. All 50 registered nurses currently assigned to the critical care units at KSSH were invited to participate. Given the specific focus on a specialized unit, the sample size was limited to the total nursing staff of the department (N = 50). A post hoc power analysis was conducted to ensure the validity of the findings. Based on the observed correlation (r = −0.699) and a significance level (α) of 0.05, the sample of 50 yielded a statistical power (1 − β) of >0.99), indicating that the sample size was sufficient to detect the identified effects. Total population sampling in a single-center tertiary setting minimizes selection bias within that institution but may limit generalizability to smaller non-specialist hospitals.

### 2.4. Participants

The target population consisted of clinical nurses in high-acuity settings. They are included provided that they are registered nurses currently employed at King Salman Specialist Hospital in the critical care unit, actively engaged in direct patient care, and have varying levels of experience and tenure in the critical care setting. Meanwhile, nurses are excluded if they are working primarily in administrative or non-clinical leadership roles. The participant flow followed a total population sampling approach. From an initial target population of 50 critical care nurses at King Salman Specialist Hospital, all 50 met the inclusion criteria and agreed to participate, resulting in a 100% response rate.

### 2.5. Data Collection Procedure

Data were collected between October and December 2024. The participants were approached by members of the research team (rather than department managers to prevent perceived coercion) during shift huddles and break times. No formal advertising was used. The written informed consent was obtained from each nurse after a detailed explanation of the study’s voluntary nature. Data were collected via paper-based surveys. Participants completed the questionnaires in a private setting and returned them to a sealed anonymous drop-box in the nursing breakroom to ensure confidentiality.

### 2.6. Instruments

This study utilized the Wong and Law Emotional Intelligence Scale (WLEIS). It is a 16-item scale that measures four subscales: Self-Emotion Appraisal, Others’ Emotion Appraisal, Use of Emotion, and Regulation of Emotion [15]. This study utilized the English version, which has been previously validated in the Saudi healthcare context [16]. In the current study, the WLEIS demonstrated high reliability with a Cronbach’s alpha of 0.89. Items are rated on a 5-point Likert scale (1 = strongly disagree to 5 = strongly agree). Total scores range from 16 to 80, with higher scores indicating greater emotional intelligence.

The second questionnaire utilized was the Job Performance Evaluation Questionnaire, adopted from Santalla-Banderali and Alvarado [17]. This scale measures job knowledge and productivity and has demonstrated a robust factorial structure across various cultural settings. In the current study, the instrument achieved a Cronbach’s alpha of 0.84. The 18-item scale is scored from 0 to 4, with higher aggregated scores indicating superior job performance. The instrument underwent English translation and was reviewed for content and clarity by a panel of experts, including one university researcher and three clinical nurse researchers.

Beyond the primary questionnaire items, the study also collected additional socio-demographic data that could act as possible confounders of the study results. These variables included age, gender, and years of experience/clinical tenure in the critical care area. These variables were assessed for an association with EI and job performance in the high-stress group.

### 2.7. Data Analysis

Statistical analysis was performed using IBM SPSS Statistics version 28.0. The analysis was conducted by the research team in consultation with a specialist biostatistician. The Shapiro–Wilk test was performed to assess data distribution. The results indicated that the data followed a normal distribution (*p* > 0.05), justifying the use of parametric tests. Descriptive statistics summarized demographic data. Point–biserial correlation (r_pb_) was used for gender (a dichotomous variable) and one-way ANOVA for age (a polychotomous variable with three categories). To determine the extent to which EI predicts JP among critical care nurses, a simple linear regression was employed.

## 3. Results

Table 1 presents the descriptive statistics between emotional intelligence and job performance. The mean score of 61.40 (out of a possible 80) suggests that critical care nurses perceive themselves as having relatively high emotional intelligence. The mean score of job performance is 52.15 (out of a possible 72), indicating a moderate-to-high level of self-perceived job performance.

The relationship between demographic categories and instrument scores was examined using point–biserial correlation. The EI (r_pb_ = 0.095; *p* = 0.514) and JP (r_pb_ = −0.089; *p* = 0.537) results suggest no significant relationship. Meanwhile, one-way ANOVA was used for age groups with EI resulting in (F = 0.945; *p* = 0.423) and JP (F = 1.120; *p* = 0.326), suggesting that gender did not significantly influence the outcomes (*p* > 0.05) (Table 2).

A simple linear regression was conducted to determine the extent to which EI predicts JP among critical care nurses. The ANOVA results demonstrate that the overall regression model was highly significant (F [1, 48] = 45.829; *p* < 0.001) (Table 3).

The regression coefficients used to evaluate the impact of EI on JP are presented below. The results indicate that EI is a highly significant predictor of performance scores (t = −6.77; *p* < 0.001) (Table 4).

## 4. Discussion

### 4.1. On EI and JP Among Critical Care Nurses

The emotional intelligence scores in this study are consistent with the findings of numerous case studies that emphasize the need for elevating EI. Emotional intelligence serves as a protective mechanism for nurses regarding burnout and turnover intention [18]. This speaks to the positive influence EI has on the nursing profession and workplace. It can be argued that emotional intelligence aids in the decision-making process for nurses [19], positively impacting job performance through a reduction in occupational stress [20]. This shows that there is a strong correlation between emotional intelligence and the performance and tenacity of critical care nurses in challenging situations. There exists variability across nursing fields when it comes to emotional intelligence. It was noted that the emotional intelligence of nurses working in intensive care units is generally higher than in general units [21]. A recent study also found a relationship between emotional intelligence and job retention among nurses [18]. This suggests that, because of the emotional demands of working in an intensive care unit, the emotional intelligence of a nurse is even more valuable, increasing their efficacy and job satisfaction.

The self-reported levels of job performance in this study tended to be high. It was reported that emotional intelligence does correlate positively with job performance. In a meta-analysis, it was shown that everyone who reported having emotional intelligence also stated that they performed their job duties to a higher standard [22]. Emotional intelligence has also been shown to improve performance at work by improving interpersonal relations and reducing conflicts [23]. Moreover, psychological well-being, which is affected by emotional intelligence, boosts job efficacy and job performance among clinical nurses [24]. In critical care, where the job responsibilities are intricate and the emotional atmosphere is high, such findings are of utmost importance.

### 4.2. Relationship Between EI, Demographics and Job Performance

Contrary to the prevailing literature, the findings demonstrated no significant relationship between EI and job performance. This result diverges from the widely established positive linkage found in previous studies conducted across various healthcare settings. For example, it was reported that nurses with higher EI exhibited superior clinical performance as they were better equipped to recognize, regulate, and apply emotions in high-pressure environments [25]. These competencies were associated with enhanced communication, stress management, and patient rapport—factors that are critical for effective care in ICUs. In contrast, our findings suggest that, within the highly demanding environment of critical care, elevated emotional sensitivity may contribute to emotional exhaustion or burnout, thereby impairing job performance. High EI levels may heighten emotional responsiveness to stressors, such as patient suffering, ethical dilemmas, or workplace tensions, potentially overwhelming even the most emotionally adept professionals and limiting their ability to function.

The socio-demographic analysis revealed no statistically significant relationship between EI and age or EI and gender. These findings contrast with those of other researchers who reported a positive correlation between demographic characteristics, such as age and experience, and enhanced emotional insight and job performance [26,27]. In our study, the absence of significant demographic effects may reflect the homogenizing influence of shared environmental stressors in critical care units. Regardless of age or gender, all nurses in this setting likely experience similar emotional and cognitive demands, which could result in a convergence of EI levels across groups. Likewise, no significant correlations were observed between job performance and age or job performance and gender. These findings align with the earlier reports that demographic variables did not meaningfully influence nursing performance [28]. However, other research has identified that younger nurses may experience higher levels of emotional exhaustion, potentially leading to diminished job performance [25]. These mixed results suggest that, while demographic variables may play a role in certain contexts, environmental and organizational factors may have a more profound impact on nursing performance. This reinforces the need for institutional strategies that emphasize psychosocial support, mentorship, and targeted training over general demographic assumptions.

The unique nature of critical care work may help to explain the divergence of this study’s results from the existing literature. High patient acuity, continuous exposure to trauma, and the need for rapid emotionally laden decision-making can impose significant psychological burdens. As noted, EI tends to have positive effects across a variety of clinical domains [29,30]. However, these benefits may not translate to the extreme conditions present in intensive care environments. The emotionally charged atmosphere, coupled with interprofessional tensions and fluctuating patient outcomes, may undermine the protective qualities of EI and instead contribute to psychological distress. This suggests that, without adequate systemic and emotional support, nurses with higher EI may become more vulnerable to emotional over-engagement, thereby diminishing their capacity for effective clinical performance.

### 4.3. Emotional Intelligence as Predictor to Job Performance Among Critical Care Nurses

Emotional intelligence (EI) is one of the most important predictors of success in a wide range of occupations. The results suggest that, in some situations where emotional intelligence grows, some performance measures may actually get worse. This suggests that some empirical conceptualizations of EI, particularly those focused on emotional control or the management of interpersonal relations, may have a detrimental effect on performance in some settings. There is a wide range of empirical support and evidence for the construct being a valid predictor of performance. Researchers argue that earlier research may have exaggerated the prediction of EI due to misinterpretation of the environment [31]. On the other hand, it was demonstrated that EI is a significant predictor of managerial effectiveness and productivity by mediating the relationship between conflict management styles and job performance in public organizations [32]. Scholars performed a meta-analysis and found that self-reported EI is a significant predictor of job outcomes, which contradicts the postulation that non-cognitive variables are the most important determinants of job performance [33].

The various studies in the literature on EI so far show its usefulness in improving employee relationships and employee management, in turn positively affecting some areas of business performance [34,35,36]. Thus, organizations should analyze whether their recruitment and selection procedures need to change in order to incorporate emotional intelligence testing in addition to cognitive testing. Higher emotional intelligence has the potential to improve interpersonal relationships and conflict management, which, in turn, should improve performance in jobs that require teamwork and collaboration. The negative correlation in this study, however, might be contextual: it might mean that some facets of emotional intelligence and performance metrics are so particular to the requirements of the job that they do not align. This should provide enough of an incentive to change how emotional intelligence is integrated within performance metrics [37,38].

The most important result of this research is the identification of an ‘emotional tax’ related to the working conditions in the nursing field’s highest-acuity areas. The relationship between EI and performance also suggests that, in the ICU’s high-stress context, the emotional strain (TMGT effect) is most likely to result in emotional exhaustion as a consequence of the trauma and grief she has to repeatedly process. Hence, it is most likely that critical cognitive resources, which allow one to remain at top technical and clinical operational levels, will be depleted. Thus, this research, as far as the context is concerned, illustrates that EI, while a robust predictor of performance, is limited in scope, and, in high-stress life-and-death situations, too much emotional sensitivity will translate into the likelihood of burnout and/or compassion fatigue.

### 4.4. Study Implications

The implications for education and recruitment in the nursing profession are also shaped by these research findings. Most importantly, these findings contradict the assumption that EI is always better and that organizations should prioritize EI in the recruitment of staff for ICUs and other similarly stressful environments. Therefore, the emphasis of training should be on the cultivation of emotional regulation and clinical detachment as opposed to emotional awareness. While emotionally attaching to patients is a professional liability for nurses, boundary setting that retains empathetic attachment creates a liability for the system that relies on the intermediate emotionally attaching clinician.

The need for the creation of specific support structures in nursing management is, for the most part, also dictated by these research findings. Since the research showed that EI predicts performance regardless of age and gender, nursing managers must analyze the group of high-EI individuals as one in need of support of a particular kind—prescribed debriefings and the assignment of alternating high- and low-stress positions. It is important for nursing managers to recognize that high-EI nurses are most likely to experience performance trade-offs by placing them in positions that create high demand for emotional input as such an understanding provides those managers with a framework for more constructive and proactive management of these employees than merely an output-focused approach.

### 4.5. Study Limitations

Despite having sufficient statistical power (1 − β > 0.99) to support the research’s findings, there are some limitations that need to be considered. The first limitation is that, because this study used a cross-sectional design within a single tertiary-level specialist hospital, it does not allow for any form of definite causal inference and therefore reduces the likelihood of generalizing the results to hospitals of a non-specialist level or to smaller healthcare settings. The second limitation is that the study relied upon self-reporting using the WLEIS and JP scales, which can result in a social desirability bias with regard to participants reporting either higher or lower than what they actually demonstrate regarding their emotional traits and productivity. Although the Job Performance Evaluation Questionnaire demonstrates cross-cultural reliability, there are no studies that have formally validated its use as an assessment of performance for either a professional or language-specific context. The questionnaire has been reviewed by an expert panel; however, based upon the absence of a formal validation study within the same population, the performance scores derived from the questionnaire may be difficult to interpret. The study accounted for both age and gender as confounders, but it did not include other confounders that could potentially affect negative correlations (resilience, burnout, amount of experience in critical care, etc.). Finally, despite the scales demonstrating a high degree of internal consistency, due to the study focusing upon a specialized highly intense nursing group within Saudi Arabia, the identified ‘emotional tax’ on performance may have been context-dependent and would require additional longitudinal studies across a wider range of clinical environments.

## 5. Conclusions

The study shows that emotional intelligence (EI) holds predictive value for job performance within the domain of critical care nursing. However, this predictive value appears to function through an inverse relationship. In particular, critical care environments demand psychological and emotional sensitivity to the point of emotional trade-offs regarding sensitivity, with practitioners potentially losing motivation and facing reduced job performance due to emotional labor. The results hold regardless of demographic characteristics as the age or gender of the respondent had no significant impact on the levels of EI or performance. The study raises important considerations about the accepted idea that “more is better” when it comes to emotional intelligence within the healthcare setting. In particular, there is an absolute need for high-EI nurses to receive support to manage the emotional engagement and the emotional labor involved.

## Figures and Tables

**Table 1:** Descriptive statistics of emotional intelligence and job performance.

Scores of WLEIS and PE	Mean (M)	Std. Deviation (SD)	Minimum	Maximum
Emotional intelligence (WLEIS)	61.4	8.12	42	78
Job performance (PE)	52.15	9.45	30	68

**Table 2:** Relationship between demographics and emotional intelligence and job performance.

Variable	Statistical Test	Emotional Intelligence	Job Performance
Gender (male vs. female)	Point–biserial (r_pb_)	r_pb_ = 0.095 (*p* = 0.514)	r_pb_ = −0.089 (*p* = 0.537)
Age group (3 categories)	One-way ANOVA (F)	F = 0.945 (*p* = 0.423)	F = 1.120 (*p* = 0.326)

**Table 3:** ANOVA results for the regression model.

Model	Sum of Squares	df	Mean Square	F	Sig.
Regression	1205.092	1	1205.092	45.829	<0.001 *
Residual	1262.188	48	26.296		
Total	2467.28	49			
Predictors: (constant), emotional intelligence; dependent variable: job performance.					

* Significant at the *p* < 0.05 level.

**Table 4:** Regression coefficients for EI predicting JP.

Model	Unstandardized B	Std. Error	Standardized Beta (β)	t	Sig.
(Constant)	37.388	2.937		12.729	<0.001
Emotional intelligence	−0.541	0.08	−0.699	−6.77	<0.001 *

* Significant at the *p* < 0.05 level.

## Data Availability

The datasets used and/or analyzed during the current study are available from the corresponding author on reasonable request in order to protect participant integrity.

## Appendix A

**Table A1:** STROBE statement—checklist of items that should be included in reports of cohort studies.

	Item No.	Recommendation	Page No.
Title and abstract	1	(a) Indicate the study’s design with a commonly used term in the title or the abstract	1
		(b) Provide in the abstract an informative and balanced summary of what was done and what was found	
Introduction			
Background/rationale	2	Explain the scientific background and rationale for the investigation being reported	2
Objectives	3	State specific objectives, including any prespecified hypotheses	3
Methods			
Study design	4	Present key elements of study design early in the paper	3
Setting	5	Describe the setting, locations, and relevant dates, including periods of recruitment, exposure, follow-up, and data collection	3
Participants	6	(a) Give the eligibility criteria, and the sources and methods of selection of participants. Describe methods of follow-up	4
		(b) For matched studies, give matching criteria and number of exposed and unexposed	
Variables	7	Clearly define all outcomes, exposures, predictors, potential confounders, and effect modifiers. Give diagnostic criteria if applicable	5
Data sources/measurements	8 *	For each variable of interest, give sources of data and details of methods of assessment (measurements). Describe comparability of assessment methods if there is more than one group	4
Bias	9	Describe any efforts to address potential sources of bias	5
Study size	10	Explain how the study size was arrived at	4
Quantitative variables	11	Explain how quantitative variables were handled in the analyses. If applicable, describe which groupings were chosen and why	5
Statistical methods	12	(a) Describe all statistical methods, including those used to control for confounding variables	5
		(b) Describe any methods used to examine subgroups and interactions	
		(c) Explain how missing data were addressed	
		(d) If applicable, explain how loss to follow-up was addressed	
		(e) Describe any sensitivity analyses	
Results			
Participants	13 *	(a) Report numbers of individuals at each stage of study—e.g., numbers potentially eligible, examined for eligibility, confirmed eligible, included in the study, completing follow-up, and analyzed	5
		(b) Give reasons for non-participation at each stage	
		(c) Consider use of a flow diagram	
Descriptive data	14 *	(a) Give characteristics of study participants (e.g., demographic, clinical, and social) and information on exposures and potential confounders	5
		(b) Indicate number of participants with missing data for each variable of interest	
		(c) Summarize follow-up time (e.g., average and total amount)	
Outcome data	15 *	Report numbers of outcome events or summary measures over time	6
6 Main results	16	(a) Give unadjusted estimates and, if applicable, confounder-adjusted estimates and their precision (e.g., 95% confidence interval). Make clear which confounders were adjusted for and why they were included	
		(b) Report category boundaries when continuous variables were categorized	
		(c) If relevant, consider translating estimates of relative risk into absolute risk for a meaningful time period	
Other analyses	17	Report other analyses done—e.g., analyses of subgroups and interactions, and sensitivity analyses	7
Discussion			
Key results	18	Summarize key results with reference to study objectives	7
Limitations	19	Discuss limitations of the study, taking into account sources of potential bias or imprecision. Discuss both direction and magnitude of any potential bias	9
Interpretation	20	Give a cautious overall interpretation of results considering objectives, limitations, multiplicity of analyses, results from similar studies, and other relevant evidence	9
Generalizability	21	Discuss the generalizability (external validity) of the study results	9
Other Information			
Funding	22	Give the source of funding and the role of the funders for the present study and, if applicable, for the original study on which the present article is based	20

* Give information separately for exposed and unexposed groups. Note: An Explanation and Elaboration article discusses each checklist item and gives methodological background and published examples of transparent reporting. The STROBE checklist is best used in conjunction with this article (freely available on the websites of PLoS Medicine at http://www.plosmedicine.org/ (accessed on 25 January 2026), Annals of Internal Medicine at http://www.annals.org/ (accessed on 25 January 2026), and Epidemiology at http://www.epidem.com/ (accessed on 25 January 2026)). Information on the STROBE initiative is available at http://www.strobe-statement.org.
